# 5-hydroxytryptamine (5-HT) reduces total peripheral resistance during chronic infusion: direct arterial mesenteric relaxation is not involved

**DOI:** 10.1186/1471-2210-12-4

**Published:** 2012-05-06

**Authors:** Robert Patrick Davis, Jill Pattison, Janice M Thompson, Ruslan Tiniakov, Karie E Scrogin, Stephanie W Watts

**Affiliations:** 1 Department of Pharmacology and Toxicology, Michigan State University, East, Lansing, MI 48824-1317, USA; 2 Department of Pharmacology and Experimental Therapeutics, Loyola University, Chicago, Stritch School of Medicine, Maywood, IL 60153, USA

**Keywords:** Serotonin (5-hydroxytryptamine, 5-HT), Blood pressure, Hypotension

## Abstract

Serotonin (5-hydroxytryptamine; 5-HT) delivered over 1 week results in a sustained fall in blood pressure in the sham and deoxycorticosterone acetate (DOCA)-salt rat. We hypothesized 5-HT lowers blood pressure through direct receptor-mediated vascular relaxation. *In vivo*, 5-HT reduced mean arterial pressure (MAP), increased heart rate, stroke volume, cardiac index, and reduced total peripheral resistance during a 1 week infusion of 5-HT (25 µg/kg/min) in the normotensive Sprague Dawley rat. The mesenteric vasculature was chosen as an ideal candidate for the site of 5-HT receptor mediated vascular relaxation given the high percentage of cardiac output the site receives. Real-time RT-PCR demonstrated that mRNA transcripts for the 5-HT_2B_, 5-HT_1B_, and 5-HT_7_ receptors are present in sham and DOCA-salt superior mesenteric arteries. Immunohistochemistry and Western blot validated the presence of the 5-HT_2B_, 5- HT_1B_ and 5-HT_7_ receptor protein in sham and DOCA-salt superior mesenteric artery. Isometric contractile force was measured in endothelium-intact superior mesenteric artery and mesenteric resistance arteries in which the contractile 5- HT_2A_ receptor was antagonized. Maximum concentrations of BW-723C86 (5- HT_2B_ agonist), CP 93129 (5-HT_1B_ agonist) or LP-44 (5-HT_7_ agonist) did not relax the superior mesenteric artery from DOCA-salt rats *vs.* vehicle. Additionally, 5-HT (10^–9^ M to 10^–5^ M) did not cause relaxation in either contracted mesenteric resistance arteries or superior mesenteric arteries from normotensive Sprague- Dawley rats. Thus, although 5-HT receptors known to mediate vascular relaxation are present in the superior mesenteric artery, they are not functional, and are therefore not likely involved in a 5-HT-induced fall in total peripheral resistance and MAP.

## Background

5-HT is a vasoactive amine synthesized in the enterochromaffin cells of the gastrointestinal tract and the raphe nucleus of the central nervous system [1,2]. The physiological actions of 5-HT are mediated by 7 major receptor subtypes (5-HT_1_- 5-HT_7_) [3]. Initially, 5-HT was identified as a potent constrictor of isolated blood vessels in the rat [4]. The primary contractile receptor in the peripheral vasculature of the rat is the 5-HT_2A_ receptor [5]. Additionally, activation of 5-HT receptors contracts human blood vessels, including: coronary arteries [6], saphenous vein, internal mammary artery [7], cutaneous hand vein [8], and pulmonary artery [9].

The constrictor effects of 5-HT initially led investigators to hypothesize that elevated levels of free plasma 5-HT, present in human and experimental models of hypertension [10,11], were acting to constrict the peripheral vasculature, resulting in elevated blood pressure. However, Diaz *et al.* demonstrated that chronic 5-HT infusion produced a sustained fall in blood pressure in the deoxycorticosterone acetate (DOCA)-salt and sham rat [12]. This suggests elevated levels of free plasma 5-HT may instead lower blood pressure. The mechanism by which chronic 5-HT infusion lowers blood pressure is not yet known. However, Diaz *et al.* further demonstrated that inhibition of nitric oxide synthase (NOS) prevented the chronic 5-HT-induced fall in blood pressure in the sham and DOCA-salt rat. This suggested an important role for NOS, and potentially nitric oxide (NO), in enabling a 5-HT-induced fall in blood pressure.

Given that a 5-HT-induced fall in blood pressure may be mediated by either a reduction in total peripheral resistance (TPR), or a reduction in cardiac output (CO), we initially sought to establish that 5-HT lowers blood pressure through a reduction in TPR. We targeted the mesenteric vasculature, which receives a large portion of cardiac output (~ 20%) as a potential site at which 5-HT may be acting to reduce TPR [13].

In addition to its well known function as a vasoconstrictor, 5-HT can also cause dilation of the peripheral vasculature. Thus, one mechanism by which 5-HT may reduce blood pressure is through direct receptor mediated vascular relaxation. Specifically, the 5-HT_2B_, 5-HT_1B_, and 5-HT_7_ receptors have been linked to dilation of peripheral arteries [14-16]. Therefore, we tested the hypothesis that 5-HT lowers blood pressure through direct receptor-mediated vascular relaxation.

We studied the 5-HT_2B_, 5-HT_1B_, and 5-HT_7_ receptors at the level of mRNA (real time PCR), protein (Western blot and immunohistochemical detection), and function (isolated contractile studies) in isolated mesenteric arteries to determine whether selective activation of these receptors was capable of direct receptor mediated vascular relaxation. We then investigated whether 5-HT was capable of relaxing a true mesenteric resistance artery. Further, we conducted experiments in both sham and DOCA-salt rats to assess potential mechanistic differences in how 5-HT lowers blood pressure.

This study provides the first evidence that chronic infusion of 5-HT reduces TPR, and that 5-HT does not act directly on the mesenteric vasculature to stimulate direct receptor mediated vascular relaxation, enabling a 5-HT-induced fall in blood pressure.

## Results

### Effects of 5-HT on CO and TPR

**Figure 1:**
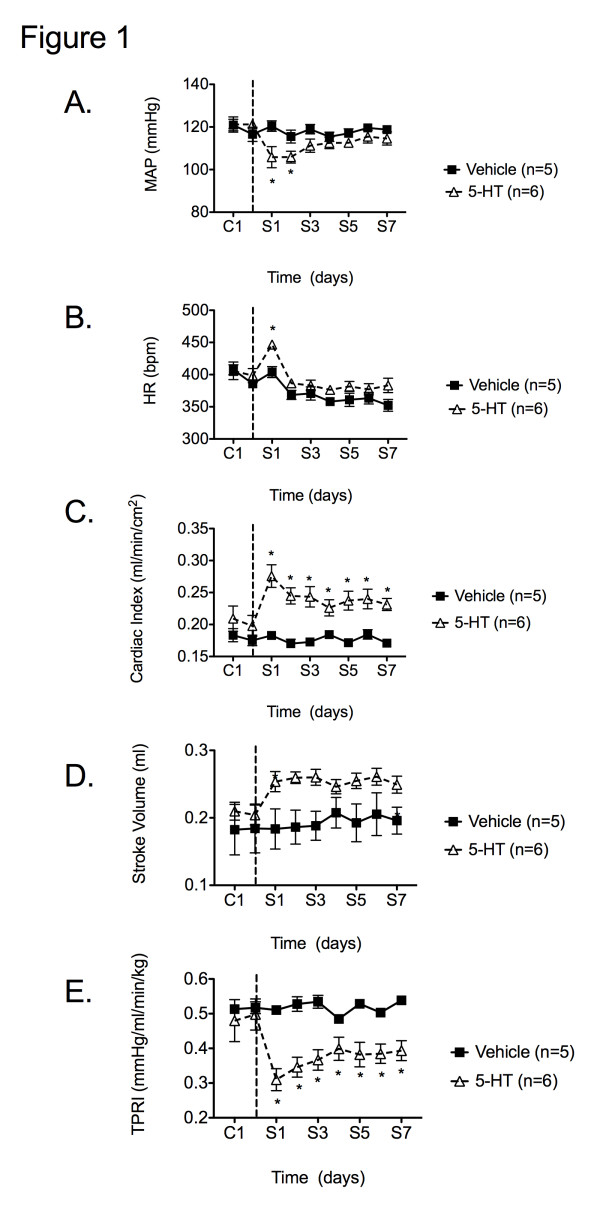
** Effect of chronic 5-HT or vehicle infusion on A) mean arterial pressure (MAP), B) heart rate (HR), C) cardiac index (CI), D) stroke volume, and E) total peripheral resistance (TPRI).** Data points are 1-hr group means +/− SEM. Number of animals per group is shown in parentheses. The vertical line denotes 5-HT or vehicle osmotic pump implant. C1 and C2 - control recordings. S1 – S7 days since initiation of 5-HT/vehicle infusion. * *P* < 0.05 vs. vehicle.

Whole animals were used to determine whether TPR (rather than cardiac output) was reduced to facilitate a 5-HT-induced fall in blood pressure. There were no between-group difference in blood pressure prior to 5-HT or vehicle infusion (C1- C2). Continuous 5-HT infusion resulted in a significant fall in MAP on the first 2 days of infusion (designated S1 and S2) (Figure 1A). Starting on day S3, MAP tended to normalize yet remained below baseline levels. 5-HT produced a significant increase in HR, cardiac index (CI) and stroke volume (SV) compared to vehicle infusion. HR returned to pre-administration levels after 1 day of infusion, while CI and SV remained significantly elevated throughout the experiment (Figure 1). Rats receiving 5-HT demonstrated a significant reduction in total peripheral resistance (TPRI; 0.31 ± 0.03 mmHg/ml/min/kg; S1) compared sto vehicle-treated animals (0.51 ± 0.01 mmHg/ml/min/kg). This effect persisted throughout the remainder of the study.

### Real time RT-PCR

**Figure 2:**
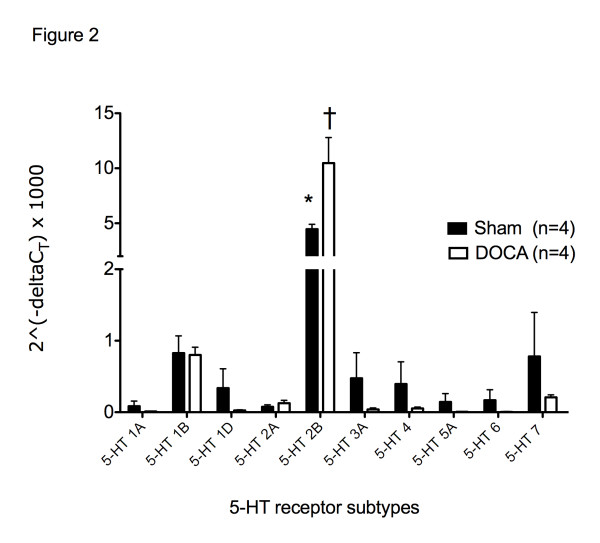
** A. mRNA expression of 5-HT receptors in the superior mesenteric artery (SMA) of sham (n = 4) and DOCA-salt (n = 4) rats using standard real time PCR analysis.** Data points represent mean ± SEM for the number of animals in parentheses. * *P* < 0.05 *vs.* 5-HT1B and 5-HT_7_ receptors. † *P* < 0.05 *vs.* sham 5- HT_2B_ receptor.

5-HT receptor transcripts were quantified using real time RT-PCR (2^-delta^ Ct x1000) in the SMA of sham and DOCA-salt rats using beta-2 microglobulin as a calibrator (Figure 2). The 5-HT_2B_ receptor mRNA was expressed at high levels in both the SMA of sham and DOCA-salt rats (4.46 ± 0.44 *vs*. 10.41 ± 2.32 respectively). 5-HT_2B_ receptor mRNA was expressed at significantly greater levels in the SMA of the DOCA-salt rat compared to the sham rat (*P* < 0.05). mRNA transcripts for the 5-HT_1B_ (DOCA-salt 0.83 ± 0.24; Sham 0.81 ± 0.11), and 5-HT_7_ receptor (DOCA-salt 0.78 ± Sham 0.62; 0.21 ± 0.04) were expressed at significantly lower levels compared to the 5-HT_2B_ receptor (*P* < 0.05) but were not different between sham and DOCA rats. The 5-HT_2B_, 5-HT_1B_, and 5-HT_7_ receptors were considered the major products and followed by Western blot, immunohistochemical detection, and contractile studies.

### Western blot analysis of the 5-HT_2B_, 5-HT_7_, and 5-HT_1B_ receptor(s)

**Figure 3:**
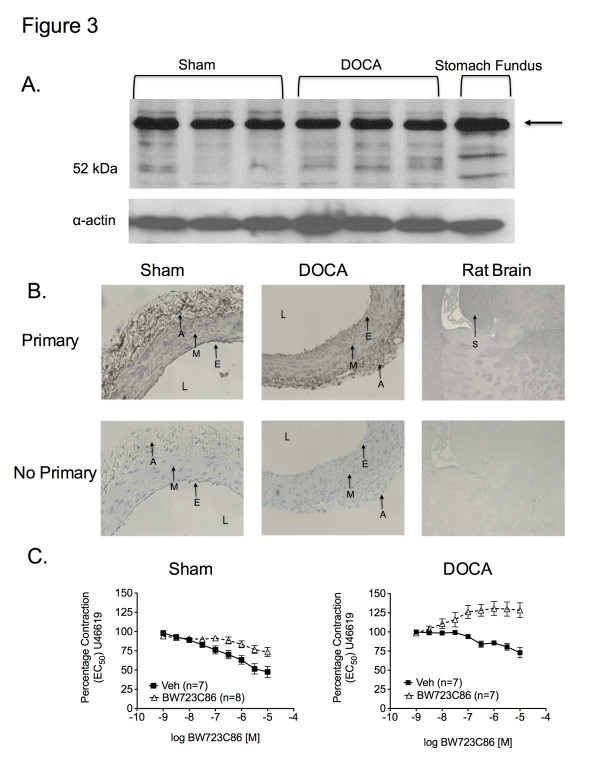
** A. Western blot of the 5-HT**_2B_**receptor in homogenates of SMA from sham (n = 6) and DOCA-salt (n = 6) rats.** Arrows = band or region of interest. **B.** Immunohistochemical staining of the 5-HT_2B_ receptor in SMA sections from sham and DOCA-salt rats. Images are sections of SMA incubated with and without primary antibody and taken with 20X objective. E = endothelium, M = media, A = adventitia, S = staining in rat brain, L = lumen (n = 6). **C.** Cumulative concentration response curve to BW723C86 *vs.* vehicle (H_2_0) in SMA contracted with U46619 in sham and DOCA-salt SMA. Data points represent mean ± SEM for the number of animals in parentheses.

**Figure 4:**
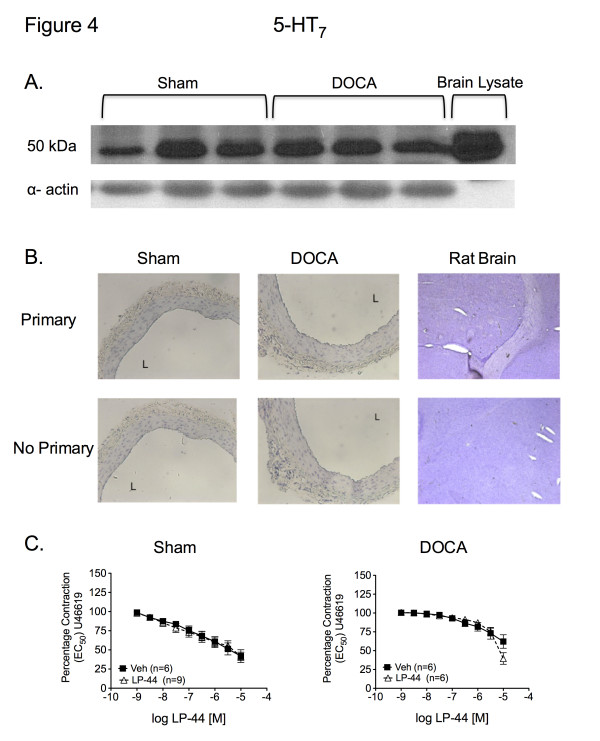
** A. Western blot analyses for identification of the 5-HT**_7_**receptor in homogenates of SMA from sham (n = 6) and DOCA-salt (n = 6) rats.** Arrows = band or region of interest. **B.** Immunohistochemical staining of the 5-HT_7_ receptor in SMA sections from sham and DOCA-salt rats. Images are sections of SMA incubated with and without primary antibody and taken with 20X objective. A = adventitia, L = lumen (n = 6). **C.** Cumulative concentration response curve to LP-44 *vs.* vehicle (DMSO) in SMA contracted with U46619 in sham and DOCA-salt SMA. Data points represent mean ± SEM for the number of animals in parentheses.

**Figure 5:**
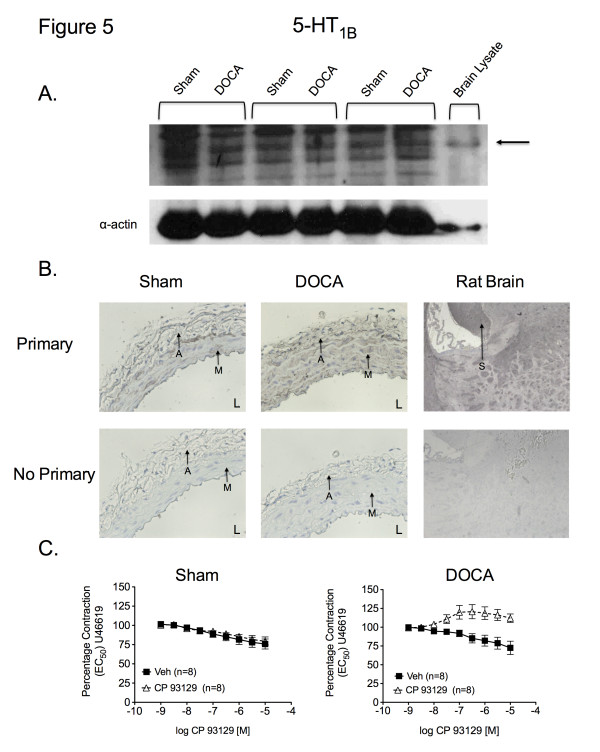
** A. Western blot analyses for identification of the 5-HT**_1B_**receptor in homogenates of SMA from sham (n = 6) and DOCA-salt (n = 6) rats.** Arrows = band or region of interest. **B.** Immunohistochemical staining of the 5-HT_1B_ receptor in SMA sections from sham and DOCA-salt rats. Images are sections of SMA incubated with and without primary antibody and taken with 20X objective. M = media, A = adventitia, S = staining in rat brain, L = lumen (n = 6). **C.** Cumulative concentration response curve to CP 93129 *vs.* vehicle (H_2_O) in SMA contracted with U46619 in sham and DOCA-salt SMA. Data points represent mean ± SEM for the number of animals in parentheses.

Antibodies directed against 5-HT_2B_ and 5-HT_7_ receptors detected bands in protein samples from SMA from sham (5-HT_2B_, 36.1 ± 3.0; 5-HT_7_, 67.6 ± 8.0 arbitrary densitometry units; respectively) and DOCA-salt rats (5-HT_2B_, 37.0 ± 2.3; 5-HT_7_, 68.8 ± 2.3 arbitrary densitometry units; respectively). The 5-HT_2B_ and 5-HT_7_ receptor antibodies also labeled protein in lanes loaded with rat stomach fundus (5-HT_2B_ control) (Figure 3A) and rat brain lysate (5-HT_7_ control) (Figure 4A), respectively. The 5-HT_2B_ receptor band was recognized at higher molecular weight in rat stomach fundus and all arterial homogenates, while the 5-HT_7_ receptor band was identified at the expected molecular weight. The 5-HT_1B_ receptor antibody revealed multiple bands in the SMA from sham (3.3 ± 1.4 arbitrary densitometry units) and DOCA-salt (4.4 ± 0.9 arbitrary densitometry units) rats. However, a band, consistent with one of the multiple bands, was also present in rat brain lysate (5-HT_1B_ control) (Figure 5A). No significant differences existed between the sham and DOCA-salt 5-HT_2B_ receptor, the sham and DOCA- salt 5-HT_7_ receptor, or the sham and DOCA-salt 5-HT_1B_ receptor expression (*P* > 0.05). However, these data demonstrated that the 5-HT_2B_, 5-HT_7_, and 5-HT_1B_ receptor(s) are present in the SMA of both sham and DOCA-salt rats.

### Immunohistochemical detection of the 5-HT_2B_, 5-HT_7_, and 5-HT_1B_ receptor(s)

Immunohistochemical detection was conducted to determine the location of 5-HT receptors within the SMA of sham and DOCA-salt rats. We observed positive staining as black/brown precipitate. 5-HT_2B_ receptor staining was observed in the endothelial, smooth muscle, and adventitial layers of SMA sections from sham and DOCA-salt rats (Figure 3B). Similar staining, corresponding to the 5-HT_1B_ receptor was observed in the media and adventitial layers (Figure 5B). Negligible 5-HT_7_ staining was observed in the adventitia. (Figure 4B). Staining was observed in the rat brain (positive control) with the 5-HT_2B_ and 5-HT_1B_ receptor antibodies, but not the 5-HT_7_ receptor antibody.

### Activation of the 5-HT_2B_, 5-HT_7_, and 5-HT_1B_ receptor(s) does not relax the superior mesenteric artery of the sham or DOCA-salt rat

Strips of SMA from sham and DOCA-salt rats were cleaned and placed in isolated tissue baths for measurement of isometric contractile force. All tissues were incubated with ketanserin (5-HT_2A_ receptor antagonist) prior to agonist concentration response curves to prevent potential contraction at high agonist concentrations (10^–5^M) and maximize the possibility of observing agonist- induced relaxation. BW-723C86 (5-HT_2B_ receptor agonist) did not relax the helical SMA strip from either sham or DOCA-salt rats compared to vehicle (Figure 3C). Instead, maximum concentrations of BW-723C86 (10^–5^M) contracted the DOCA-salt SMA (128 ± 9.7% of half-maximal U46619-induced contraction) compared to vehicle (73 ± 6.6%) (*P* > 0.05). Similarly, neither the 5-HT_7_ receptor agonist LP-44 nor the 5-HT_1B_ receptor agonist CP 93129 (Figure 4C and Figure 5C, respectively) induced a concentration-dependent relaxation above that observed with vehicle in U46619-contracted mesenteric arteries from sham or DOCA-salt rats. Finally, sodium nitroprusside (10^–6^M) completely relaxed U46619-contracted mesenteric arteries following treatment with BW-723C86 (0.26 ± 0.21% of half-maximal U46619-induced contraction), LP-44 (1.2 ± 0.54%), and CP 93129 (1.5 ± 0.92%).

### 5-HT does not relax mesenteric resistance vessels or the superior mesenteric artery in normotensive rats

**Figure 6:**
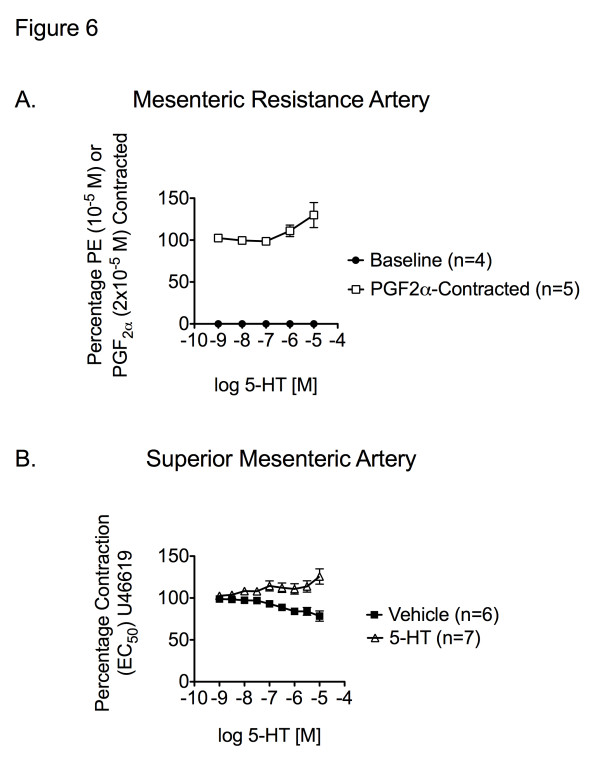
** A. Cumulative concentration response curve to 5-HT generated from baseline, and contracted with prostaglandin F**_2_**alpha (PGF**_2α_**) in the presence of ketanserin in the normal superior mesenteric resistance artery.B.** Cumulative concentration response curve to 5-HT *vs.* vehicle (H_2_O) in SMA contracted with U46619 and incubated with ketanserin in SMA. Data points represent mean ± SEM for the number of animals in parentheses.

Because the above studies were negative, we next investigated whether 5-HT was capable of relaxing a true mesenteric resistance artery. Endothelial-intact mesenteric resistance vessels (200 micron diameter) were mounted in a wire myograph chamber for measurement of isometric contractile force. 5-HT (10^–9^M - 10^–5^M) contracted the isolated mesenteric resistance vessel from baseline and when contracted with prostaglandin F_2_ alpha (PGF2α) and incubated with ketanserin (Figure 6A). 5-HT (10^–9^M - 10^–5^M) also contracted the isolated SMA when contracted with U46619 and incubated with ketanserin (Figure 6B). These same resistance vessels relaxed over 50% to ACh (10^–6^M) and forskolin (1 µM). Relaxation to 5-HT was not observed under any condition. These findings suggest that 5-HT alone does not relax the superior mesenteric resistance vessels.

## Discussion

The main objective(s) of this study were to: 1) determine whether 5-HT lowers blood pressure through a reduction in total peripheral resistance (TPRI) or cardiac output (CO), 2) identify the primary 5-HT receptors expressed in the superior mesenteric artery (SMA) of sham and DOCA-salt rats, and 3) determine whether activation of these receptors relaxes SMA of sham or DOCA-salt rats.

### 5-HT reduces total peripheral resistance in sham and DOCA-salt rats

5-HT infusion produced a significant reduction in MAP and TPRI, supporting our original hypothesis that 5-HT lowers blood pressure through direct receptor mediated vascular relaxation. These data confirm and extend our previous findings that chronic elevations in free circulating 5-HT reduce MAP, and increase HR over the course of a 1 week 5-HT infusion [12]. TPR is reduced throughout the duration of the infusion, despite a recovery in blood pressure, a novel finding. The recovery of blood pressure may be due to the persistent elevation in SV and CI. HR is only transiently increased and therefore raises the question of whether 5-HT is also acting directly on the heart to increase SV. 8-OH-DPAT (5-HT_1A_ and 5-HT7 receptor agonist) produces a sustained increase in cardiac output (CO) following hemorrhagic shock [17]. Thus, activation of the 5- HT_1A_ receptor may explain why blood pressure recovers despite a persistent reduction in TPR. Ultimately, by defining these important hemodynamic parameters (HR, SV, CI and TPR), we were able to pursue a careful investigation of the vasculature as a potential site of action for a 5-HT-induced fall in blood pressure. The mesenteric vasculature was selected as the potential site of action for 5-HT due to the large portion of cardiac output it receives (~20%) [13]. We hypothesized that 5-HT may be acting on mesenteric vasculature to reduce TPR and lower blood pressure.

### 5-HT does not directly relax the mesenteric arterial vasculature

The 5-HT_2B_ receptor was a leading candidate in our investigation due to its location on the endothelial cell, reports of its ability to stimulate NO release [18], and the absolute dependence of the 5-HT-induced depressor response on nitric oxide synthase [12]. Bordoff *et al.* demonstrated that treatment with either a 5-HT_2B_ receptor antagonist or an inhibitor of NOS abolished a bypass-induced fall in blood pressure in the male normotensive Wistar rat [14]. Bordoff *et al.* hypothesized 5-HT released from mechanically disrupted platelets, contributed to falling blood pressure in patients placed on cardiopulmonary bypass. This suggested 5-HT may activate NOS, and potentially release NO. The 5-HT_1B_ and 5-HT_7_ receptors were also considered leading candidates in our investigation. Calama *et al..* demonstrated that intra-arterial administration of L- 694, 247 (5-HT_1B/1D_ receptor agonist) produced vasodilation in the hindquarter of the anesthetized rat [15]. Further, activation of the 5-HT_7_ receptor is associated with vascular smooth muscle relaxation, and a long-lasting depressor response in the anesthetized rat [16,19,20]. These studies suggest 5-HT may be acting at either the 5-HT_1B_, or 5-HT_7_ receptor(s) to lower blood pressure.

RT-PCR, Western blot, and IHC analysis identified expression of the 5-HT_2B_, 5-HT_7_, and 5-HT_1B_ receptors in the SMA of sham and DOCA-salt rats. The 5-HT_7_ receptor antibody recognized protein in SMA homogenates from sham and DOCA-salt rats, but the same antibody did not elicit a strong immunohistochemical stain in sections of SMA from either sham or DOCA-salt rats. Furthermore, we also failed to demonstrate adequate staining in brain sections using multiple other antibodies against the 5-HT_7_ receptor. Nonetheless, RT-PCR and Western blot data strongly support the existence of the 5-HT_7_ receptor in the SMA of sham and DOCA-salt rats.

Despite compelling evidence that 5-HT_2B_, 5-HT_7_, and 5-HT_1B_ receptors are expressed in the mesenteric vascular bed, no significant relaxation to their respective 5-HT receptor agonists was observed in either the SMA of the sham or DOCA-salt rat. Instead, activation of the 5-HT_2B_ receptor (*via* BW723C86) and 5-HT_1B_ (*via* CP93129) caused contraction of the SMA of the DOCA-salt rat. These results are consistent with previous studies in the DOCA-salt aorta, which demonstrated that activation of the 5-HT_2B_ and 5-HT_1B_ receptor(s) in the DOCA- salt aorta elicits a contractile response [21,22].

Interestingly, a significant body of evidence suggests opposing roles for the 5-HT_2B_ receptor depending on its location within the vasculature (endothelial cell vs. smooth muscle), and/or underlying pathology (presence or absence of hypertension) [21,22]. For example, the 5-HT_2B_ receptor is up-regulated, and contractile in the aorta of the DOCA-salt rat *vs.* sham rat [22]. This evidence may explain why BW723C86 contracted the SMA of DOCA-salt rat, but did not contract the SMA of the sham rat. Finally, 5-HT itself did not relax either a true mesenteric resistance vessel or the SMA in the presence of ketanserin (a 5-HT_2A_ receptor antagonist). We hypothesized that the presence of ketanserin might reveal or unmask a relaxant response in the mesentery. Thus, we must conclude that the 5-HT_2B_, 5-HT_7_, and 5-HT_1B_ are not involved in relaxing the SMA and 5-HT is not likely acting directly in the mesenteric vasculature to mediate a 5-HT- induced fall in TPR and blood pressure.

### Potential mechanism(s) underlying a 5-HT-induced fall in blood pressure

Several possibilities may explain a 5-HT-induced fall in blood pressure. First, 5- HT may be acting in a different vascular bed, rather then the mesentery, to release nitric oxide (NO), reduce TPR, and lower blood pressure. Direct receptor- dependent vascular relaxation has been observed in the rat jugular vein, the porcine pulmonary artery, the canine coronary artery, and the human umbilical artery [23-27]. Further, previous studies also suggest an important role for the serotonin transporter (SERT) in mediating a 5-HT-induced fall in blood pressure [28]. Thus, we cannot rule out the possibility that 5-HT may be taken up *via* SERT at an alternate site (vascular), leading to nitric oxide synthase (NOS) activation and NO release.

The skeletal muscle vasculature, and the cutaneous vasculature are alternative sites at which 5-HT may be acting to reduce TPR, and lower blood pressure [28,29]. For example, fluoxetine (5-HT reuptake inhibitor) dilates isolated skeletal muscle arterioles [30]. Additionally, Alsip *et al.* demonstrated 5-HT is capable of dilating the rat skeletal muscle arteriole [31]. The skeletal muscle vasculature receives a large portion of cardiac output (~ 40%), such that changes in resistance could account for large changes in blood pressure [13]. The cutaneous vasculature is also a potential site at which 5-HT may be acting to reduce TPR, and lower blood pressure, but this is more controversial. 5-HT causes a significant increase in tail temperature in the rat, suggestive of heat loss through cutaneous vasodilation, and a fall in blood pressure [32,33]. Similarly, the 5-HT releaser fenfluramine is associated with cutaneous dilation in the conscious rat [34]. Low doses of intra- arterially infused 5-HT significantly increased human forearm blood flow in healthy volunteers [35]. Collectively, these data suggest 5-HT may relax the cutaneous vasculature, reduce peripheral resistance, and lower blood pressure. However, the association between changes in cutaneous blood flow and systemic change(s) in TPR needs to be studied further.

Second, 5-HT may reduce vascular tone by inhibiting sympathetic nerve activity (SNA) rather than directly causing relaxation. 5-HT_1B/1D_ receptors expressed on the presynaptic sympathetic nerve terminals inhibit norepinephrine release, an action that would decrease vascular tone [36]. 5-HT may also act centrally to inhibit SNA. Recent studies demonstrated 5-HT is capable of crossing the blood brain barrier *via* the serotonin transporter (SERT) [37]. At present, we cannot rule out the possibility that 5-HT acts centrally to lower blood pressure.

## Conclusions

We recognize several limitations of the present study. First, it is possible that alternate 5-HT receptor(s) (identified by PCR) are expressed and functional in the SMA, causing direct receptor mediated vascular relaxation. However, none of these receptor(s) have been as strongly linked to vascular relaxation as those chosen for the current study. Finally, we also acknowledge that only one 5-HT receptor agonist was used to investigate each 5-HT receptor (5-HT_2B_, 5-HT_1B_, 5-HT_7_) of interest. The fact that 5-HT itself did not relax the “unmasked”, antagonized vessel, supports that the mechanism of direct relaxation does not exist in the mesenteric vasculature. In summary, this study provides the first evidence that 5-HT reduces blood pressure through a reduction in total peripheral resistance during a chronic infusion. However, the reduction in TPRI is not likely due to activation of the 5-HT_2B_, 5-HT_7_, and 5-HT_1B_ receptors in the SMA of sham or DOCA-salt rat.

## Methods

### Animals

The Michigan State University and Loyola University Chicago, Stritch School of Medicine Institutional Animal Care and Use Committees (IACUC) approved all protocols. Male Sprague Dawley rats (225–250 g, Charles River Laboratories) were used. Hemodynamic studies [measurement(s) of mean arterial pressure (MAP), heart rate (HR), cardiac output (CO), stroke volume (SV), and calculations of cardiac index (CI) and total peripheral resistance] were completed in conscious freely moving Sprague Dawley rats. Isolated tissue experiments (RT-PCR, Western blot, immunohistochemistry, and isolated contractile experiments) were performed in the SMA from sham and DOCA-salt rats.

### DOCA-salt hypertension

Sprague–Dawley rats underwent left uninephrectomy, and a DOCA pellet was implanted subcutaneously (200 mg/kg). Sham rats underwent left uninephrectomy, but were not implanted with DOCA pellet. Rats were given standard rat chow *ad libitum*. DOCA-salt water was supplemented with 1% NaCl and 0.2% KCl for the duration of the study. Sham rats received tap water for the duration of the study. Hypertension was established after 4 weeks of initiation of DOCA. Sham systolic blood pressure was < 140 mmHg, while DOCA-salt systolic blood pressure was > 140 mmHg measured by tail cuff method.

### Anesthesia and analgesia

All rats were anesthetized with isoflurane (2% in 100% O_2_) and ventilated mechanically. At the time of surgery, the incision site was treated with bupivicaine. Incisions were closed with silk suture. Rats were treated with amoxicillin (150 mg/kg/i.m.) following surgery and 3 days thereafter. All rats were treated with rimadyl (5 mg/kg) and buprenorphine (35 µg/kg) for 2 days for general analgesia.

### Surgical methods: alzet osmotic pump

A small incision was made at the base of the neck. Blunt dissection was used to create a small subcutaneous pocket between the scapulae. Alzet Osmotic Pumps (Model 2ML1, Duret Corporation, Cupertino, CA, 10 µL/hr 7 days) were loaded with 5-HT creatinine complex (25 µg/kg/min) dissolved in 1 M HCl, (normalized to pH 7 by 4 M NaOH) and inserted under the skin.

### Surgical methods: ascending aortic flow probe

Rats were given an injection of antisialogogue, glycopyrrolate (4 mg/kg, s.c.), then anesthetized with isoflurane, intubated and mechanically ventilated. An ultrasonic transit-time flow probe (model 3SB; Transonic Systems Inc., Ithaca, NY) was placed around the ascending aorta through an incision in the third intercostal space. The thoracic incision was closed in layers, and the lungs were reinflated with negative pressure. The probe cable was tunneled s.c. and externalized at the nape of the neck and secured with a silastic cuff.

### Surgical methods: blood pressure probe

During the same surgery, radiotelemetery probes (C50-PXT, Data Science International, St. Paul, MN, USA) were implanted to enable chronic blood pressure recording. The left femoral artery was exposed through a groin incision and ligated distally. The tip of the probe catheter was advanced through a hole made in the artery and advanced into the abdominal aorta. The body of the probe was placed subcutaneously on the left flank. All rats recovered for 10 days prior to the start of data collection. Mean arterial pressure (MAP), heart rate (HR), and ascending aortic blood flow (i.e. CO) were recorded daily, between 10:00 am and 2:00 pm while the rats rested unrestrained in their home cage. Recordings began at least 60 min for connection to the blood flow meter, after which MAP and HR were recorded continuously for 1 hr. Total peripheral resistance (mmHg/ml/min/kg) and cardiac Index (CI) were normalized to body mass. Where CI = CO / (9.1 x g^0.66^).

Where g = body mass in g, (9.1 x g^0.66^) = body surface area in cm^2^, and TPRI = CI/MAP.

### Tissue preparation

Rats were anesthetized with pentobarbital (60 mg/kg i.p.) and the superior mesenteric artery or mesenteric resistance artery was removed and placed in physiological salt solution (PSS) containing (M): NaCl 130; KCl 4.7; KH_2_PO_4_ 1.8; MgSO_4_ * 7H_2_O 1.7; NaHCO_3_ 14.8; dextrose 5.5; CaNa_2_EDTA 0.03, CaCl_2_ 1.6 (pH 7.2).

### Real-time RT-PCR

Superior mesenteric artery was removed and placed in sterile water, then cleaned of fat and blood. Total RNA was isolated using the MELT Total Nucleic Acid Isolation System and reverse transcribed with Superscript II reverse transcriptase (Invitrogen, Carlsbad, CA). Standard real-time RT-PCR was done using a GeneAMP 500 Real-Time PCR machine (Applied Biosystems, Carlsbad, CA) and SYBR Green PCR Master Mix (Applied Biosystems). Rat primers were purchased from SABiosciences (Frederick, MD): 5-HT_1A_ (RefSeq Accession #: NM_012585.1; 191 bp amplicon), 5-HT_1B_ (RefSeq Accession #: NM_022225.1; 103 bp amplicon), 5-HT_1D_ (RefSeq Accession #: NM_012852.1; 173 bp amplicon), 5-HT_2A_ (RefSeq Accession #: NM_017254.1; 191 bp amplicon), 5-HT_2B_ (RefSeq Accession #: NM_017250.1; 140 bp amplicon), 5- HT_3A_ (RefSeq Accession #: NM_024394.2; 179 bp amplicon), 5-HT_4_ (RefSeq Accession #: NM_01285.31; 83 bp amplicon), 5-HT_5A_ (RefSeq Accession #: NM_013148.1; 154 bp amplicon), 5-HT_6_ (RefSeq Accession #: NM_024365.1; 186 bp amplicon), 5-HT_7_ (RefSeq Accession #; NM_022938.2; 99 bp amplicon), and calibrator control (beta-2 microglobulin) (RefSeq Accession #: NM_012512, 128 bp amplicon). PCR conditions were: 95°C for 10 minutes followed by 40 cycles of (95°C, 15 sec; 60°C, 60 sec). A standard dissociation curve was run following the above cycle conditions. Each sample was run in duplicate. No template controls (NTC) were run for each primer set as well as no Reverse Transcriptase (no RT) samples to verify that the primers themselves did not amplify and that no genomic contamination had occurred with RNA isolation, respectively.

### Western blot analysis

Superior mesenteric arteries were cleaned, frozen, and then ground into a powder. Homogenation buffer (125 mM Tris (pH 6.8), 4% SDS, 20% glycerol, 0.5 mM phenylmethylsulfonyl fluoride, 1 mM orthovanadate, 10 µg/ml aprotinin, 10 µg/ml leupeptin) was added and the homogenates were vortexed briefly. Protein concentration was determined with the BCA protein kit (Sigma, catalog #BCA1). Western analysis of rat SMA homogenates (50 µg) was performed and proteins transferred to PVDF (5-HT1B and 5-HT7) or nitrocellulose (5-HT2B). Blots were then incubated overnight at 4°C with 5-HT_1B_ (1 µg/ml; Abcam, Cambridge, MA; Catalog # ab13896), 5-HT2B (1:1000; BD Pharmingen, San Diego, CA; Catalog # 556334), or 5-HT7 (1:1000; Abcam, Cambridge, MA; Catalog # ab13898). Following 5-HT receptor antibody incubation, blots were reprobed for smooth muscle α-actin (1:2000; EMD Chemicals/Calbiochem, Gibbstown, NJ) to ensure equal protein loading. All blots were developed using species-specific HRP- conjugated secondary antibodies and ECL reagents (Amersham/GE Healthcare Life Sciences, Piscataway, NJ).

### Immunohistochemistry

Slides containing sections of paraffin-embedded rat superior mesenteric artery were dewaxed, unmasked using Unmasking Solution (Vector Laboratories, Burlingame, CA) and taken through a standard protocol. Slides were incubated with 5-HT_1B_, 5-HT_2B_ or 5-HT_7_ antibody (5 µg/ml) (same antibodies as used for Western blots) in 1.5% blocking serum. Slides were washed in phosphate buffered saline and incubated with a peroxidase-conjugated secondary antibody in 1.5% blocking serum for 30 minutes, followed by a 30 min incubation in Vectastain Elite ABC Reagent (Vector Laboratories). 3,3-diaminobenzidine/H_2_O_2_ was applied until staining appeared (1–4 minutes). The slides were counterstained with hematoxylin (Vector Laboratories, Burlingame, CA).

### Isolated tissue bath

Endothelium-intact SMA were cleaned and cut into helical strips for measurement of isometric contractile force. SMA strips were mounted in warmed (37°C) and aerated (95% O_2_, 5% CO_2_) tissue baths (30 ml PSS) on Grass isometric transducers (FT03; Grass instruments, Quincy, MA, USA), connected to an ADInstruments PowerLab (ADInstruments, Colorado Springs, CO). Tissues were placed under optimal resting tension (600mg; previously determined) and allowed to equilibrate for 1 hr before an initial challenge with a maximal concentration of phenylephrine (10^–5^M). After the initial challenge, tissues were washed until tone returned to baseline. Then, a half-maximal concentration of phenylephrine (10^–7^M) was added to the bath, followed by acetylcholine (ACh; 10^–6^M) to determine the integrity of the endothelial layer. Tissues that relaxed more than >50% in ACh were considered to have intact endothelium. Tissues were washed until they returned to baseline. Strips were incubated with ketanserin (50 nM; 5-HT_2A_ antagonist) for 15 minutes to prevent activation of the 5-HT_2A_ receptor. Tissues were then contracted half-maximally to U46619 (thromboxane A_2_ agonist) (~30 min incubation with ketanserin). Cumulative concentration-response curves were generated for each of the following agonists: 5-HT *vs.* vehicle (H_2_O), BW-723C86 (5-HT_2B_ agonist) *vs.* vehicle (H_2_O), CP 93129 (5-HT_1B_ agonist) *vs.* vehicle (H_2_O), and LP-44 (5-HT_7_ agonist) *vs.* vehicle (DMSO). At the end of each experiment, sodium nitroprusside (SNP) (10^–6^M) was added to demonstrate that each SMA was able to relax under experimental conditions set forth.

### Wire myograph

Under a stereomicroscope with a calibrating eyepiece and in cold PSS, two tungsten wires were inserted through the lumen of a cleaned mesenteric resistance artery (~200-250 micron diameter). One wire was attached to an isometric force transducer with a detection range of 0.002-10g, while the other was connected to a micrometer-attached support. Force generation was recorded on a Grass Model 7D polygraph. The dual chamber, kept at 37°C and perfused *via* a peristaltic pump with warmed and oxygenated PSS, allowed mounting of two parallel vessels. A passive tension of ~400 mg (appropriate for generating optimal force in a resistance artery) was applied. Vessels were allowed to equilibrate for 1 hr prior to initial challenge with PE (10^–5^M). After initial washout, experiments were performed as described above for isolated tissue bath.

### Statistical analysis

For *in vivo* data analysis, between group differences were assessed by a two- way ANOVA with repeated measures followed by post hoc testing using Bonferroni’s procedure (GraphPad Prism 5). For isometric contractile studies, relaxation is reported as a percentage of initial contraction to a half-maximal concentration of U46619 (thromboxane A_2_ agonist). Repeated measures two- way ANOVA followed by the Bonferroni post hoc test was used to compare concentration-response curves. An unpaired Students *t-*test was used to compare differences in maximal response(s) between agonist *vs.* vehicle. In all cases, *p* < 0.05 was considered significant. All results are presented as the mean ± SEM.

## Competing interests

The authors declare that they have no competing interests.

## Authors’ contributions

RPD: performed contractile experiments, analyzed data, is primary author of the manuscript; JP: performed IHC and Western experiments, analyzed data, contributed to paper, read final version; JMT: performed IHC and Western experiments, analyzed data, contributed to paper, read final version; RT: performed whole animal flow experiments, analyzed data, contributed to paper, read final version. KES: performed whole animal flow experiments, analyzed data, contributed to paper, read final version. SWW: performed myography experiments, analyzed data, contributed to paper, read final version, coordinated the study. All authors read and approved the final manuscript.
